# Disruption of Mitophagy‐Related Gene Expression in Gestational Diabetes Mellitus: A Transcriptomic and Machine Learning Approach

**DOI:** 10.1155/jdr/7913374

**Published:** 2026-03-24

**Authors:** Souhaib Bouati, Shaima Ameen, Nour al dain Marzouka, Fadwah Alhantoobi, Emad Masuadi, Ghada Mohammed, Noha Ahmed Mousa, Shahad Mahmoud, Muhieddine Seoud, Hisham Mirghani, Halima Alnaqbi, Ayman Pathan, Maha Saber-Ayad, Wael Osman

**Affiliations:** ^1^ Department of Biological Sciences, College of Medicine and Health Sciences, Khalifa University, Abu Dhabi, UAE, kustar.ac.ae; ^2^ Department of Genetics and Genomics, College of Medicine and Health Sciences, United Arab Emirates University, Al Ain, UAE, uaeu.ac.ae; ^3^ College of Medicine and Health Sciences, Institute of Public Health, United Arab Emirates University, Al Ain, UAE, uaeu.ac.ae; ^4^ Department of Clinical Sciences, College of Medicine, University of Sharjah, Sharjah, UAE, sharjah.ac.ae; ^5^ Department of Obstetrics and Gynecology, Sheikh Shakhbout Medical City (SSMC), Abu Dhabi, UAE, ssmcabudhabi.ae; ^6^ Department of Foetal Medicine Services, NMC Royal Hospital, Abu Dhabi, UAE; ^7^ Department of Biomedical Engineering and Biotechnology, College of Medicine and Health Sciences, Khalifa University, Abu Dhabi, UAE, kustar.ac.ae; ^8^ Center for Biotechnology, Khalifa University, Abu Dhabi, UAE, kustar.ac.ae; ^9^ Sharjah Institute for Medical Research, University of Sharjah, Sharjah, UAE, sharjah.ac.ae; ^10^ Department of Pharmacology, Faculty of Medicine, Cairo University, Cairo, Egypt, cu.edu.eg

**Keywords:** ATF4, GDM, gene expression, mitophagy, MUL1, PINK1, placenta, TOMM7

## Abstract

**Background:**

Gestational diabetes mellitus (GDM) is a pregnancy‐associated metabolic disorder linked to adverse maternal and fetal outcomes. Mitochondrial dysfunction is a recognized feature of GDM, yet the role of mitophagy—the selective degradation of damaged mitochondria—remains insufficiently understood.

**Objective:**

This study examined the expression and regulatory patterns of mitophagy‐related genes (MRGs) in GDM using publicly available transcriptomic datasets.

**Methods:**

Transcriptomic datasets available in public repositories were analyzed to explore MRG expression and regulatory dynamics in GDM. RNA‐seq data from two datasets: GSE203346 (placental and cord blood samples) and GSE154414 (placental samples) were analyzed to identify differentially expressed mitophagy genes. Additionally, maternal circulating blood RNA‐seq data from GSE154377 were included for machine learning analysis. These datasets, which encompassed samples collected across multiple trimesters, facilitated a comparative evaluation of MRG expression dynamics in both placental tissue and maternal blood throughout pregnancy. A curated list of 65 MRGs was evaluated using edgeR and DESeq2 for differential expressions (DEs). Temporal expression dynamics were modeled with the multiclassPairs package in R using GSE154377.

**Results:**

Consistent downregulation of four critical MRGs—MUL1, PINK1, TOMM7, and ATF4—was observed in GDM placental tissue (GSE154414) and in both placental tissue and fetal umbilical cord blood (GSE203346) but not in maternal peripheral blood. In healthy pregnancies, these genes exhibited distinct temporal regulation across gestation, a pattern disrupted in GDM. Classifier models based on MRG expression accurately predicted gestational stage in controls (accuracy > 85%) but performed poorly in GDM (accuracy < 50%). Functional enrichment analyses revealed impaired mitochondrial protein import, autophagy, and oxidative stress responses.

**Conclusion:**

These findings suggest that mitophagy dysregulation is an early and persistent defect in GDM, with MUL1, PINK1, TOMM7, and ATF4 emerging as potential biomarkers and therapeutic targets. The results support the hypothesis that mitochondrial quality control failure contributes to the pathogenesis of GDM with similar patterns shown in both placental and cord blood tissues. However, these genes were not significantly altered in plasma, highlighting tissue context as a critical factor in detecting mitophagy‐related dysregulation.

## 1. Introduction

Gestational diabetes mellitus (GDM) is a prevalent metabolic disorder during pregnancy, defined by glucose intolerance that either originates or is first identified in this period [1]. It is estimated that hyperglycemia in pregnancy affects ~16.7% of pregnancies worldwide, with 80% due to GDM [2]. However, the prevalence varies based on diagnostic criteria, ethnic background, and population‐specific risk factors [2–5]. In regions such as the United Arab Emirates, where advanced maternal age, obesity, and insulin resistance syndromes are increasingly prevalent, the burden of GDM is particularly significant [6, 7]. Beyond its immediate complications—such as fetal overgrowth, hypertensive disorders, and increased cesarean delivery rates—GDM is associated with long‐term risks for both mother and child, including type 2 diabetes mellitus (T2DM), metabolic syndrome, and cardiovascular disease [8–10].

The pathophysiology of GDM involves an imbalance between the physiological rise in insulin resistance during late pregnancy and the pancreatic β‐cells’ inability to sufficiently augment insulin secretion. In a typical pregnancy, insulin sensitivity naturally decreases to ensure adequate glucose availability for fetal development [11]. This is usually counterbalanced by increased β‐cell proliferation and insulin production. However, when this compensatory mechanism fails—due to β‐cell dysfunction or excessive insulin resistance—GDM develops [12–15].

Mitochondria are central to these processes, as they regulate energy production, oxidative stress, apoptosis, and calcium homeostasis, all of which are critical for insulin secretion and sensitivity [16, 17]. Research has shown that mitochondrial dysfunction in the placenta and maternal tissues contributes to impaired β‐cell function, defective insulin signaling, and heightened oxidative stress in GDM [15, 18–20]. This dysfunction is often characterized by elevated levels of reactive oxygen species (ROS), reduced mitochondrial membrane potential, and altered mitochondrial DNA (mtDNA) expression profiles [21, 22]. As a result of these mitochondrial stress states, coordinated transcriptional responses and cellular turnover occur, resulting in the release of stress‐responsive RNA species into the bloodstream. Accordingly, serum cell‐free transcriptomic profiling can capture alterations in the expression of genes related to mitochondrial stress‐response pathways and mitochondrial mitophagy as a noninvasive indicator of mitochondrial dysfunction.

Mitophagy, the process of autophagic clearance of damaged or dysfunctional mitochondria, plays a key role in maintaining mitochondrial quality control and metabolic balance. It is regulated by pathways such as the PINK1/Parkin axis, mitochondrial fission and fusion events, and receptor‐mediated degradation (e.g., through MUL1 or BNIP3). Dysregulation of mitophagy leads to the accumulation of defective mitochondria, exacerbating cellular stress, inflammation, and metabolic dysfunction [23–25].

While mitophagy has been extensively studied in contexts such as neurodegeneration and aging, its role in pregnancy and gestational metabolic adaptations remains underexplored. Recent animal studies suggest that impaired mitophagy in placental and adipose tissues contributes to insulin resistance and fetal overgrowth [26, 27]. However, human studies are limited, and no comprehensive analysis of selective mitophagy regulation distinct from general mitochondrial dysfunction or bulk autophagy across placental and maternal tissues throughout pregnancy has been conducted in GDM.

Current studies highlight associations between genetic and epigenetic factors, mtDNA mutations, and autophagy regulators, but most lack direct functional validation or mechanistic insight into how these alterations contribute specifically to mitochondrial quality control through mitophagy in GDM. For instance, the role of SNPs and epigenetic modifications in regulating mitophagy‐specific pathways remains incompletely explored, and the functional consequences of mtDNA mutations in GDM are poorly characterized [28]. Moreover, the contribution of mitophagy regulators such as p66Shc and their impact on trophoblast function under high glucose conditions are not well understood [29, 30]. Importantly, existing human GDM placenta studies have primarily examined canonical autophagy markers and general indicators of mitochondrial dysfunction, with few systematically interrogating mitophagy‐specific regulators or alternative pathways beyond the classical PINK1–Parkin axis. These gaps highlight the demand for integrative transcriptomic analyses across maternal and placental tissues to provide a clearer understanding of mitophagy dysregulation in GDM and to explore their potential as biomarkers or therapeutic targets.

This study addresses this gap by analyzing transcriptomic data from multiple high‐quality RNA‐sequencing datasets to evaluate mitophagy‐related gene (MRG) expression in GDM. Using two independent placental datasets (GSE203346 and GSE154414) and a longitudinal maternal blood dataset (GSE154377) spanning all three trimesters, we sought to clarify how MRGs are disrupted across tissues and over gestation. Beyond maternal metabolic health, GDM has significant implications for offspring, who face increased risks of obesity, glucose intolerance, and type 2 diabetes later in life, with growing evidence of sex‐specific vulnerability, particularly among female offspring. These long‐term effects are increasingly linked to epigenetic mechanisms, including DNA methylation and other chromatin modifications established in utero, which may encode a molecular “memory” of the GDM environment and contribute to the intergenerational transmission of metabolic risk [31–33]. By focusing on the placenta and cord blood, our study targets the maternal‐fetal interface and fetal circulation, which are key compartments through which mitophagy dysregulation could influence such epigenetic programming and long‐term metabolic trajectories.

## 2. Materials and Methods

### 2.1. Dataset Selection

Three RNA‐sequencing datasets, publicly accessible through the Gene Expression Omnibus (GEO) database, were chosen for analysis. The first dataset, GSE203346 (combined sample size = 92), comprises placental transcriptome profiles from individuals with GDM and healthy controls, generated using the Illumina HiSeq platform. The second dataset, GSE154414 (combined sample size = 8), includes placental samples from GDM and control pregnancies, sequenced on the NovaSeq 6000 platform. Together, these datasets facilitated cross‐platform validation of placental gene expression. The third dataset, GSE154377, consists of maternal blood (plasma) transcriptomes obtained during the three trimesters of pregnancy from GDM and healthy individuals, utilizing the Illumina HiSeq 2500 system. This longitudinal dataset supported the examination of mitophagy gene expression patterns throughout gestation. The serum circulating cell‐free RNA (cfRNA) (GSE154377) was used to complement placental RNA‐seq in a translational, longitudinal manner. This dataset enabled the assessment of whether placental mitophagy dysregulation is reflected at a systems level in a clinically accessible biofluid, the characterization of trimester‐specific trajectories of MRG sets, and the evaluation (using machine learning) of whether coordinated mitophagy gene expression patterns can differentiate pregnancy state (GDM versus control and/or gestational stage), even though individual genes are not significantly differentially expressed. However, these analyses are exploratory in nature, while our primary mechanistic conclusions are based on the transcriptomic analysis of placental and cord blood. Machine learning analyses of placental RNA‐seq datasets were not appropriate because of the small sample size, class imbalance, and heterogeneity, which are likely to result in overfitting and a lack of generalizability.

### 2.2. Gene Curation for Mitophagy

A list comprising 65 genes associated with mitophagy (MRGs) was developed through the integration of the Gene Ontology (GO) term GO:0061726 (mitophagy), Kyoto Encyclopedia of Genes and Genomes (KEGG) pathway databases, and the inclusion of genes identified in recent studies focusing on mitophagy and mitochondrial quality control. Figure S1 illustrates the entire gene curation process through KEGG. Following integration across datasets, the final list comprises 58 genes when multiple datasets are combined. This curated list features notable regulators and is detailed in Table S1.

### 2.3. Preprocessing and Normalization

The samples from the datasets (GSE203346 and GSE154414) were normalized using both DESeq2’s variance‐stabilizing transformation (VST) and the trimmed mean of M‐values (TMM) method from edgeR. For the longitudinal blood dataset (GSE154377), TPM‐normalized values were directly applied. Quality control and normalization diagnostics, such as PCA plots and dispersion estimates, were performed in R (version 4.2.1).

### 2.4. Differential Expression (DE) Analysis

DE analysis was carried out independently for each dataset, comparing GDM and control samples, using DESeq2 (v1.38.3) and edgeR (v3.42.4). Significant differentially expressed genes were identified based on an adjusted *p*‐value (FDR) < 0.05 and an absolute log2 fold change > 0.4. The EnhancedVolcano package was employed for visualizing DE results and presented as volcano plots.

### 2.5. Functional Enrichment Analysis

Using clusterProfiler package in R, we ran enrichment (BP/CC/MF via enrichGO) and KEGG enrichment on downregulated MRGs (IDs mapped with org.Hs.eg.db/bitr). The background was all expressed genes, and *p*‐values were adjusted by Benjamini–Hochberg with an FDR < 0.05. Autophagy, mitochondrial dynamics, and cellular stress responses were the most enriched categories.

### 2.6. Protein–Protein Interaction (PPI) Network Analysis

We generated a PPI network for significantly downregulated MRGs (Benjamini–Hochberg FDR < 0.05, |log_2_FC| ≥ 0.58) using STRING v11.5 (*Homo sapiens*, all evidence channels enabled) with a minimum interaction score = 0.700, and disconnected nodes were hidden. Cytoscape v3.10 was used to visualize the network (nodes/edges with STRING combined score as edge weight). Using cytoHubba, we cross‐validated hub genes using betweenness centrality and maximal clique centrality (MCC); genes ranked highly by at least two metrics were retained and presented as full node/edge tables and hub ranking figures.

### 2.7. Trimester‐Based Expression Analysis

Temporal expression dynamics of MRGs were evaluated in GSE154377 (maternal blood‐serum RNA‐seq). Raw counts were merged across GSMs, matched to curated sample metadata, and restricted to normal and GDM pregnancies. We constructed a DESeqDataSet (DESeq2 package, R) with design = ~condition, using raw integer counts. Genes with low overall expression (row sums < 10) were excluded prior to model fitting. A median‐of‐ratios approach was used to estimate size factors, and DESeq2 defaults were followed for dispersion, outlier detection (Cook’s distance), and NB GLM fitting. The DE of GDM and normal was calculated with Wald tests (…, contrast = c(“condition”, “GDM”, “Normal”), with *p*‐values adjusted by Benjamini–Hochberg FDR (*α* = 0.05). The log_2_ fold changes were estimated using shrinkage (e.g., lfcShrink(type = “apeglm”)).

We displayed expression values as per‐gene distributions across trimesters and conditions using ggplot2 with variance‐stabilized values (VST; blind = FALSE); normalized counts were used when appropriate.

As an optional stage‐wise analysis, we reran DESeq2 within each trimester (1st, 2nd, 3rd, and delivery) with the same prefiltering and design = ~condition; FDR control was applied across all trimester‐specific tests.

### 2.8. Machine Learning Classification of Trimester Status

The trimester classification problem was framed as a supervised k‐Top Scoring Pairs (kTSP) problem using multiclassPairs package in R. The analyses were limited to 1st–3rd trimesters and carried out separately within controls and within GDM to avoid confounding by disease status. Starting from MRGs present in both cohorts (Entrez→Ensembl via biomaRt), we applied VST on counts (blind = TRUE), filtered features with filter_genes_TSP (featureNo = 100 and UpDown = TRUE), trained one‐vs‐rest TSP models with train_one_vs_rest_TSP, and obtained predictions using predict_one_vs_rest_TSP.

We assessed the model using standard metrics (accuracy, precision, recall, and F1). Given the small, uneven samples and lack of evaluation data, this is an exploratory analysis to see if mitophagy gene patterns retain trimester‐specific structure in GDM versus controls, not a diagnostic test.

### 2.9. Visualization and Statistical Analysis

All analyses were run in R (Version 4.2.1). DE was performed with DESeq2 (design = ~condition), using the median‐of‐ratios size‐factor normalization, Wald tests, Benjamini–Hochberg FDR correction (*α* = 0.05), and log_2_ fold‐change shrinkage (apeglm). The main contrast was GDM vs. normal. The figures were created using ggplot2 (boxplots of VST values, blind = FALSE), EnhancedVolcano (|log_2_FC| ≥ 0.58 and FDR ≥ 0.05), and pheatmap/ComplexHeatmap (row‐scaled *Z*‐scores, Euclidean distance, and complete linkage).

In the trimester classification, we used multiclassPairs (Top Scoring Pairs and one‐vs‐rest) on VST data after gene filtering with filter_genes_TSP (featureNo = 100 and UpDown = TRUE). Due to the absence of cross‐validation or a hold‐out set, performance was summarized from in‐sample (resubstitution) predictions (accuracy, macro/weighted precision, recall, and F1). A complete list of packages and analysis tools can be found in Table S2.

## 3. Results

### 3.1. Placentas From Patients With GDM Show DEs of Mitophagy Genes

Sixty‐one genes were initially curated from the KEGG pathway database to focus our analysis on mitophagy‐related processes, which were subsequently consolidated into 58 unique genes. The genes that regulate mitophagy include ubiquitin‐dependent regulators, E3 ligases, receptor‐mediated mitophagy proteins, mitochondrial fission/fusion regulators, autophagy components, and stress‐sensitive transcription factors as presented in Figure S1. A DESeq2 analysis of the placental and cord blood RNA‐seq dataset GSE203346 and GSE154414 (combined sample size = 92; GSE154414 alone *n* = 8) identified four MRGs as downregulated in GDM in comparison to controls. These genes are PINK1, TOMM7, ATF4, and MUL1. Table 1 reports the exact statistics (log_2_FC and adjusted *p*‐values), and Figure 1A shows the volcano plot. Detailed results of the whole MRGs (*n* = 58) can be found in Table S1. Interestingly, the direction of effect is consistent with reports in similar metabolic and pregnancy disorders (e.g., T2D and preeclampsia), suggesting mitochondrial dysfunction is common to both. We also present gene expression patterns as heatmaps, with Figure 1B showing expression patterns for the top four significant genes, while Figure S2 shows results for all mitophagy genes. Detailed descriptions of these key genes and their biological functions in mitophagy and mitochondrial health can be found in Table S3.

Figure 1Differences in the expression of mitophagy‐related genes between gestational diabetes mellitus (GDM) and the control (NEG) groups. (A) The volcano plot, in which blue highlights indicate genes significantly downregulated in GDM (FDR < 0.05 and log_2_FC < −0.4). These include MUL1, PINK1, ATF4, and TOMM7. (B) A heatmap illustrates genes (rows) compared with samples (columns), and each cell is colored according to its expression level. The columns are grouped by GDM vs. control, and values are transformed and rescaled (*Z*‐scores) to make patterns comparable. A cooler color indicates a lower expression, a warmer color, and a higher expression; hierarchical clustering groups genes and samples with similar profiles together. The figures show data derived from the analysis of placental‐derived datasets GSE203346 and GSE154414.

**Figure:**
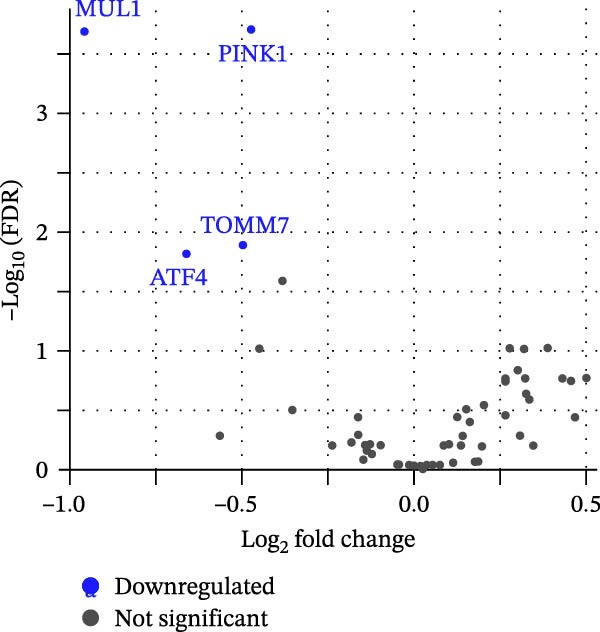
(A)

**Figure:**
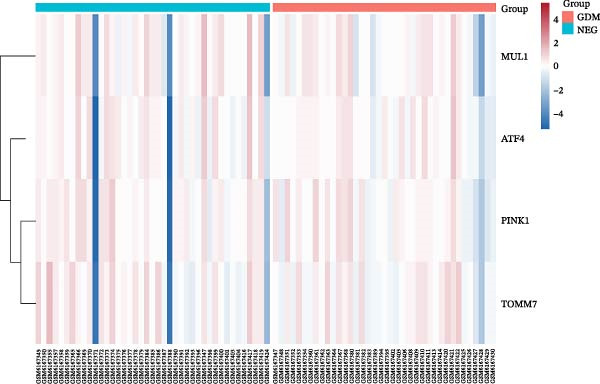
(B)

**Table 1:** Identified key mitophagy genes that were significantly downregulated in placental tissues from GDM patients and controls.

ID (Entrez)	Gene symbol	logFC ^∗^	logCPM ^∗^	*p*‐Value ^∗^	FDR ^∗^
79594	*MUL1*	−0.955	10.719	4.82E−06	2.08E−04
65018	*PINK1*	−0.472	13.446	6.94E−05	2.01E−04
54543	*TOMM7*	−0.496	13.182	6.75E−04	1.30E−02
468	*ATF4*	−0.659	14.690	1.06E−03	1.53E−02

^∗^Statistics include log_2_ fold change (logFC), log counts per million average in all samples (logCPM), raw *p*‐values, and false discovery rate (FDR). Significant genes with FDR < 0.05 and logFC < −0.4 were considered downregulated.

### 3.2. Enrichment Analysis Indicates Autophagy and Mitochondrial Dysfunction

The functional enrichment of the downregulated MRGs (clusterProfiler) was dominated by GO terms related to mitochondrial organization, oxidative stress response, and autophagy regulation, as well as KEGG pathways connected to insulin, AMPK, and FoxO signaling (Figure S3A–C). The transcriptome analysis of placental tissue and maternal blood tissues revealed that all genes displayed similar functional interactions during enrichment analysis. In GDM, these pathways correlate with established mechanisms (insulin resistance, oxidative stress, and placental dysfunction). MRGs are consistently downregulated, suggesting impaired pathway activation rather than compensatory mechanisms. These relationships are illustrated with pathway visualizations such as KEGG/ClueGO, highlighting both insulin signaling and the novel enrichment of mitophagy/autophagy. Accordingly, GDM is associated with decreased mitophagy and mitochondrial quality control processes, and mitochondrial quality control could potentially be an underexplored factor in this disease.

### 3.3. PPI Network Highlights Central Hub Genes

In STRING‐derived PPI networks of downregulated MRGs, TOMM7, MUL1, and PINK1 were identified as hub genes (high degree centrality), implicating core regulators of mitochondrial dynamics and mitophagy (Figures S4, S5). For easier interpretation and a more structured representation of networks, interactions were grouped by biological process (e.g., mitochondrial fission regulators and ubiquitin ligases). In addition to being implicated in GDM, these hub genes are implicated across metabolic conditions, highlighting their broader biological relevance and potential for use as biomarkers and therapeutics (Table S4). Based on their hub positions and functional importance, they may serve as promising biomarker candidates for early detection of GDM or as therapeutic targets.

### 3.4. Trimester‐Specific MRG Expression Patterns Are Altered by GDM

**Figure 2:**
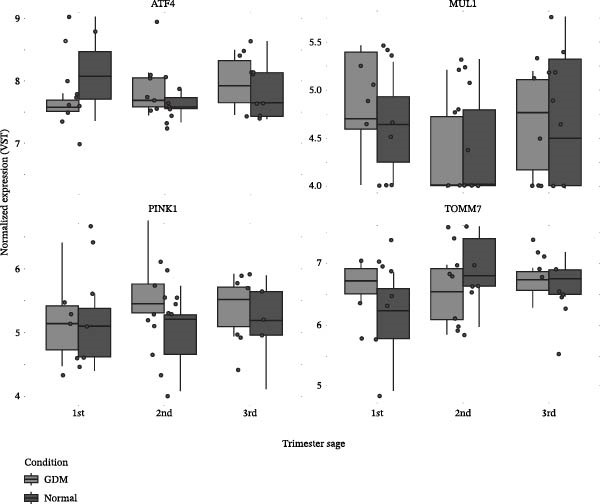
The expression of mitophagy regulators (ATF4, PINK1, MUL1, and TOMM7) across pregnancy in GDM compared to normoglycemia. GDM is represented by light gray boxes, while normal pregnancies are represented by dark gray boxes (see inset legend). Trimester‐ and condition‐specific regulation patterns are highlighted by expression values. The expression values were preprocessed and VST‐normalized as described in Section 2. This figure shows data derived from the analysis of maternal plasma‐derived dataset GSE154377.

Controls showed dynamic, trimester‐specific trajectories for PINK1, TOMM7, ATF4, and MUL1, with expression rising and falling across stages and forming clear peaks (Figure 2). These patterns appeared blunted or irregular in GDM. Based on this impression of group differences, the 3rd‐trimester subset showed modest and nonsignificant differences (Figure 2). Table S4 provides statistical comparisons across trimesters and groups based on variance‐stabilized (VST) values.

### 3.5. Machine Learning Classifiers Show Trimester Classification Degrades in GDM

MulticlassPairs classifiers trained on control samples accurately predicted the trimester (>90%; Figure S6, Table S4). A confusion matrix showing similar or superior accuracy to controls was found in the GDM subset. Nevertheless, this estimate is not directly comparable since the GDM subset contained fewer samples, which can inflate multiclass accuracy. The probability and margin plots for GDM trimesters indicate reduced separability despite nominal accuracy (Figures S6). In the absence of cross‐validation or a held‐out test set, we believe that results should be interpreted as exploratory, using VST‐normalized expression in blood serum rather than placental tissue.

## 4. Discussion

This study provides evidence that the expression of key MRGs is significantly dysregulated in GDM, disrupting both basal expression levels and temporal regulation. Transcriptomic analyses of placental tissue and maternal blood revealed the downregulation of four MRGs including PINK1, TOMM7, ATF4, and MUL1. These genes play pivotal roles in mitochondrial quality control and stress adaptation. In particular, the identification of MUL1 dysregulation in GDM is novel, highlighting its potential as a new biomarker or therapeutic target.

The intergenerational aspect of GDM reinforces our emphasis on placental and cord blood compartments. Offspring exposed to GDM in utero, especially female children, face a higher risk of later obesity and diabetes, with emerging evidence suggesting epigenetic modifications as a mechanism linking the intrauterine environment to long‐term metabolic disease [32–34]. Changes in placental transcriptomics and mitophagy‐related disturbances may drive this programming by affecting nutrient supply, inflammatory signaling, and oxidative stress, while cord blood represents the fetal response to these signals. Therefore, studying mitophagy‐related signatures in the placenta and cord blood is not only important for understanding maternal disease mechanisms but also for exploring how GDM might establish a lasting metabolic risk profile in the next generation.

**Figure 3:**
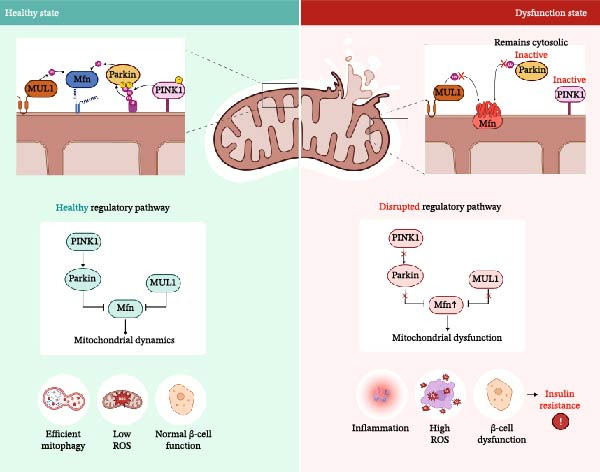
A parallel mitophagy control system using PINK1–Parkin and MUL1 and its disruption in GDM. Left: In physiological conditions, PINK1 accumulates on damaged mitochondria and recruits/activates Parkin, while MUL1 ubiquitinates and restrains mitofusin (Mfn). Combined, these axes promote mitochondrial dynamics and mitophagy, maintaining low ROS and normal β‐cell function. Right: A transcriptomic analysis of GDM suggests that PINK1, Parkin, and MUL1 are downregulated, which results in Mfn accumulation (↑Mfn), failure of Parkin activation (remains cytosolic/inactive), and impaired PINK1 signaling. The resulting mitochondrial dysfunction reduces mitophagy and increases ROS, leading to inflammation and insulin resistance. Mfn, mitofusin; ROS, reactive oxygen species.

As shown in Figure 3, the four genes highlighted in this study are fundamental for the mitophagy pathway within mitochondria. MUL1 (also referred to as Mulan, MAPL, GIDE, and HADES) is an E3 ubiquitin ligase located on the mitochondrial outer membrane (OMM) that regulates mitochondrial dynamics, mitophagy, and cellular metabolism. Through the ubiquitination of mitochondrial substrates, including mitofusin 2 (MFN2), MUL1 facilitates mitochondrial fission, a critical step for the selective elimination of damaged mitochondria via autophagy. Importantly, MUL1 not only functions within classical mitophagy pathways but also serves as a compensatory mechanism capable of operating independently of the canonical PINK1/Parkin‐mediated mitophagy under stress conditions (Figure 3).

Our study confirms previous research linking impaired mitophagy to insulin resistance and metabolic stress in diabetic patients [35–37]. It has been shown that dysfunction of TOMM7, a key regulator of mitochondrial import and PINK1 stabilization, can impair mitochondrial turnover and exacerbate oxidative stress [38, 39]. Similarly, the loss of ATF4, which controls mitochondrial protein homeostasis, indicates mitochondrial dysfunction [40, 41]. However, in our case, ATF4 exhibits divergent behavior, downregulated in placental datasets and reported as upregulated in liver and β‐cell stress models. Taking this into account, tissue‐specific regulation is possible, which needs to be explored further. Our results can also be compared with those from other metabolic diseases or pregnancy‐related disorders to provide a broader perspective on the disease.

It is interesting to note that samples from the datasets (GSE203346 and GSE154414) showed consistent MRG downregulation. There was, however, an important nuance revealed by machine learning analysis of longitudinal serum data (GSE154377). In control pregnancies, classifiers were able to correctly predict trimesters, while, in GDM, the nominal accuracy was similar or higher. There is a lack of held‐out testing in this comparison due to the small sample size and uneven trimester coverage within the GDM subset, and the comparison cannot be made directly due to the imbalance between classes and the small sample size. Additionally, GDM may also dampen dynamic transcriptomic adaptation throughout pregnancy; this hypothesis is supported by our temporal expression plots (Figure 3) [42–44]. It is important to consider differences in tissue source (placenta or serum) when interpreting patterns across data sets. As a result of the consistency between placental datasets, even though sample sizes differ, our findings are considered reliable.

Based on regulatory network modeling and motif enrichment analyses (Supporting Information: Supplementary Methods), the observed dysregulation may be attributed to the suppression of transcriptional regulators, such as FOXO3, NRF2, and ATF4. Previously, these factors were reported to be associated with diabetes and metabolic syndrome as well as stress response, autophagy, and mitochondrial biogenesis [41, 45–49]. It can be speculated that reduced ATF4 activity in GDM is partially responsible for the downregulation of MUL1 and PINK1, while FOXO3 and NRF2 may further exacerbate mitochondrial dysfunction as a result of the suppression of MRGs.

Although MUL1, PINK1, TOMM7, and ATF4 show widely consistent downregulation in placental tissue and cord blood, it is not established that impaired mitophagy will have the same effects on all compartments. In the placenta, mitochondrial dynamics and quality control are closely related to nutrient transport, steroidogenesis, vascular remodeling, and oxidative stress, and mitochondrial dysfunction in GDM has been associated with fetal overgrowth and later cardiometabolic risk in offspring [50]. In fetal cord blood, altered expression of MRGs may reflect changes in circulating immune and hematopoietic cells, inflammatory tone, and early metabolic set points—mechanisms that may contribute to long‐term neurobehavioral and metabolic outcomes after maternal diabetes [10, 51]. In maternal blood, where transcript levels are modest and often statistically insignificant, mitophagy‐related changes may constitute a systemic “echo” of mitochondrial stress in maternal organs and the placenta, with relevance as a circulating biomarker rather than a primary driver of pathology. Accordingly, the conserved mitophagy signature in placentas and cord blood samples represents a disruption in mitochondrial quality control superimposed on distinct tissue functions, resulting in possible differences in downstream phenotypes across the two compartments.

Lastly, the absence of statistically significant changes in these key mitophagy genes in maternal cfRNA should not be interpreted solely as evidence of tissue specificity. Several nonexclusive factors may explain this observation. First, cfRNA is highly fragmented, present in low abundance, and derived from multiple maternal and fetal tissues. This can dilute placenta‐specific transcriptional signals and reduce sensitivity for detecting modest expression changes. Second, cfRNA primarily reflects dynamic cell turnover and transcript release rather than steady‐state intracellular expression, making it less suitable for capturing dysregulation of tightly regulated cell‐intrinsic processes such as mitophagy. Third, timing effects may play a role: Placental mitophagy impairment may occur earlier or more transiently during gestation than during the blood sampling window in the serum dataset, leading to temporal misalignment between tissue pathology and circulating signals. Finally, technical preprocessing choices, such as VSTs and conservative multiple‐testing correction, may further attenuate small fold changes in serum data. Together, these considerations suggest that the lack of DE in maternal blood reflects the biological and technical constraints of circulating transcriptomics rather than the absence of mitophagy dysregulation in GDM.

Clinically, impaired mitophagy may contribute to fetal overgrowth, disruptions in nutrient transport, and maternal insulin resistance in GDM [35, 50, 52]. Mitophagy disruption, therefore, may not only be viewed as a secondary characteristic but may also contribute to the pathology of GDM. However, causality cannot be inferred from bulk, cross‐sectional datasets, and impaired mitophagy may both contribute to and reflect metabolic and inflammatory stress. These genes have the potential to become promising biomarkers and therapeutic targets if they are further explored. Since DEs were established in placental tissue and longitudinal analysis was conducted in blood serum, clinical translation will require prospective, gestational age–matched blood‐based validation (e.g., cfRNA or extracellular‐vesicle assays) and evidence of specificity beyond general metabolic stressors. A PPI network analysis revealed that TOMM7, PINK1, and MUL1 form interconnected hubs regulating mitophagy, further supporting their diagnostic potential. Since the network is derived from curated interactomes, it should be regarded as hypothesis‐generating rather than pregnancy‐specific, and functional confirmation is warranted in trophoblast/placental models. There are some limitations to this study, including its reliance on bulk RNA‐seq data, lack of single‐cell resolution, and lack of functional validation or maternal‐fetal paired analyses. Future studies should address limitations such as protein‐level validation (e.g., Western blot) and functional assays. There are also dataset‐specific constraints: The trimester analysis was performed on blood‐serum RNA rather than placenta tissue, limiting direct comparison across datasets and possibly attenuating placenta‐specific mitophagy signals. In this study, sample sizes were modest, which reduced power and complicated direct comparison of accuracy between groups. Finally, although mitochondrial and mitophagy dysfunction are increasingly linked to diabetes complications in multiple tissues, including heart, skeletal muscle, adipose tissue, and β‐cells, the precise clinical consequences of the transcriptomic changes we describe cannot be inferred from cross‐sectional data alone [53]. Longitudinal follow‐up of children born from GDM pregnancies, with integrated assessment of mitochondrial function, epigenetic marks, and metabolic outcomes, will be essential to determine how compartment‐specific mitophagy alterations in placenta, cord blood, and maternal blood translate into concrete disease trajectories across the life course [51].

In the pan‐organ atlas, OXPHOS was consistently suppressed across maternal tissues, a metabolic signature commonly related to mitochondrial stress [54]. In response to such stress, PINK1 usually stabilizes on the outer mitochondrial membrane, which triggers mitophagy; however, our finding that PINK1 is markedly downregulated suggests that this canonical quality control pathway may be impaired in GDM. In light of this, it is possible that, even though mitochondrial stress levels are elevated, the machinery required to activate mitophagy is simultaneously suppressed, potentially compromising mitochondrial quality control.

Based on the results of the AJOG study, mitochondrial translation and respiratory chain pathways are strongly suppressed in placentas from patients with GDMA1, GDMA2, and T2DM [55]. By identifying the loss of key regulators of mitophagy (PINK1), mitochondrial dynamics (MUL1), protein import (TOMM7), and the integrated stress response (ATF4), our data suggest that impaired mitochondrial quality control may be an upstream contributor to the pathway‐level mitochondrial deficits described in their study. The convergent findings highlight mitochondrial stress as a unifying axis of diabetic placental pathology and provide gene‐level information that complements and extends previous multiomic observations.

In the plots and classifiers, VST counts were used, which improve homoscedasticity but can degrade dynamic range, alter gene rankings within samples, and obscure very small fold changes. Furthermore, class imbalance and stage coverage affect classifier metrics; higher nominal accuracy in the GDM subset should be interpreted cautiously, as estimates can be inflated by sparse or uneven class structure and, if patient‐level dependence is not fully controlled in cross‐validation, by nonindependence of samples. It was not possible to eliminate residual confounding or batch effects due to the lack of uniformly available clinical covariates (e.g., BMI, treatment, and specific sampling windows). Lastly, the results in this paper, generated through bioinformatics and machine learning approaches, should be considered exploratory and require cross‐validation as they are generally descriptive rather than predictive. Despite these constraints, the consistency of results across placental datasets, supported by network integration, enhances the biological credibility of mitophagy impairment in GDM but emphasizes the need for larger, balanced longitudinal cohorts, proteomic validation of single cells, and paired maternal–placental analysis.

The use of spatial transcriptomics, metabolomics, and CRISPR‐based manipulations of key MRGs in placental organoids or animal models may provide further insights into causality and therapeutic potential. It may also be possible to uncover unique genetic or environmental influences on mitophagy regulation in GDM by expanding analyses to population‐specific cohorts.

## 5. Conclusion

In this multitissue, cross‐dataset analysis, GDM cases were consistently associated with downregulation and temporal disruption of four critical genes MUL1, PINK1, TOMM7, and ATF4 in transcriptomic profiling of placental and maternal blood samples. As a result of these findings, we suggest that impaired mitophagy is associated with insulin resistance, oxidative stress, and metabolic dysfunction involved in the pathogenesis of GDM. These genes may serve as biomarkers with potential predictive value for early diagnosis and as targets for therapeutic interventions aimed at enhancing mitochondrial resilience during pregnancy.

## Author Contributions

Wael Osman secured the funding for this project, oversaw the manuscript corrections, and supervised the entire project. Souhaib Bouati and Shaima Ameen conducted the data extraction and analysis and drafted the initial manuscript. Wael Osman, Maha Saber‐Ayad, Ghada Mohammed, Noha Ahmed Mousa, Shahad Mahmoud, Muhieddine Seoud, and Hisham Mirghani contributed to the study design and manuscript editing. Emad Masuadi provided guidance for the statistical analysis and contributed to the study design. Nour al dain Marzouka supervised the machine learning data analysis and developed the multiclassPairs package in R. Halima Alnaqbi and Fadwah Alhantoobi contributed to the data analysis, with Fadwah Alhantoobi performing the PPI and STRING models. Ayman Pathan conducted the revision of the manuscript with Souhaib Bouati.

## Funding

This research was funded by the Khalifa University of Science and Technology through the Faculty Start‐up Funding under Project ID KU‐INT‐FSU‐2020‐36.

## Disclosure

All authors reviewed and revised the manuscript and approved its submission.

## Conflicts of Interest

The authors declare no conflicts of interest.

## Supporting Information

Additional supporting information can be found online in the Supporting Information section.

## Supporting information


**Supporting Information** A Word document containing these supporting items is provided alongside the manuscript. The supporting information are described below in brief: (I) Supplementary Methods: The Supplementary Methods outline the advanced analyses conducted to support the primary results. We begin by validating trimester‐specific classifiers through repeated, stratified subsampling (100 iterations) to evaluate sampling stability beyond cross‐validation, reporting mean and SD values for accuracy, recall, and class‐probability margins. Next, we conduct unsupervised coexpression analysis of mitophagy genes (using Ward.D2/Euclidean clustering) and visualize the results with heat maps to identify trimester‐ and condition‐specific patterns. Temporal gene dynamics are modeled using generalized additive models (GAMs) across gestational age to capture nonlinear expression trends (e.g., PINK1, ATF4, and TOMM7). Upstream regulatory mechanisms are investigated through iRegulon motif enrichment, which predicts transcription factors targeting downregulated mitophagy genes, with TF→target networks visualized for clarity. Crosstalk analyses (using crosstalkR and KEGG graphs) examine intersections between mitophagy pathways and insulin/metabolic‐stress signaling. The mitochondrial localization of key genes is verified using the Human Protein Atlas. Lastly, batch effects are assessed (via SVA and PCA), confirming no significant batch‐related variance prior to downstream analyses. (II) Supporting Figures: Figure S1. A KEGG schematic of the mitophagy pathway is used as a reference framework. Figure S2. A heat map showing the expression levels of mitophagy‐related genes in placental samples (GDM versus control), pooled from GSE203346 and GSE154414 (*n* = 92). Highlighted are key downregulated genes (PINK1, TOMM7, ATF4, and MUL1). Figure S3. A GO enrichment analysis of MUL1 interactors using ClueGO: bar charts (biological processes and molecular functions) and pie charts (category distribution); the terms used emphasize ubiquitination/ISGylation and protein modification pathways. Figure S4. The functional enrichment (GO terms/pathways) of the integrated protein–protein interaction network incorporating targets and their interactors. Figure S5. STRING PPI subnetworks for (A) MUL1, (B) TOMM7, (C) PINK1, and (D) ATF4, with node colors reflecting GO functional categories. Figure S6. MulticlassPairs trimester classification performance for GSE154377: separate models trained within control (A) and GDM (B) groups after variance‐stabilizing transformation; feature selection based on one‐vs‐rest (top‐100; UpDown = TRUE). (III) Supporting Tables: Table S1. Analysis of mitophagy‐associated genes and differential expression statistics (Entrez ID, symbol, logFC, logCPM, *p*‐value, and FDR), including downregulated MUL1, PINK1, TOMM7, and ATF4. Table S2. Bioinformatics resources and software used (purpose and source), including GEO, edgeR, ggplot2, pheatmap, Cytoscape/ClueGO, BioGRID, biomaRt, and org.Hs.eg.db. Table S3. Detailed descriptions of key mitophagy genes (official name, symbol, gene ID, chromosomal location, and concise functional summary for MUL1, PINK1, TOMM7, and ATF4). Table S4. The performance metrics for trimester classifiers on GSE154377 (control vs. GDM) are accuracy, macro/weighted precision, recall, and F1; control models show high accuracy, while GDM models achieve perfect agreement in this dataset.

## Data Availability

Data are openly available in a public repository that issues datasets with DOIs. Repository URL: https://www.ncbi.nlm.nih.gov/geo/. Repository name: Gene Expression Omnibus (GEO). Reference Numbers: GSE203346, GSE154414, and GSE154377.

## References

[1] Care Diabetes , Care in Diabetes—2022, Diabetes Care. (2022) 45, no. Supplement_1, S17–S38, 10.2337/dc22-S002. 34964875

[2] Atlas D. , International Diabetes Federation, IDF Diabetes Atlas, 2015, 7th edition, International Diabetes Federation.

[3] Duncan B. B. , Magliano D. J. , and Boyko E. J. , IDF Diabetes Atlas 11th Edition 2025: Global Prevalence and Projections for 2050, 2025, Oxford University Press. 10.1093/ndt/gfaf177 40874767

[4] Yuen L. and Wong V. W. , Gestational Diabetes Mellitus: Challenges for Different Ethnic Groups, World Journal of Diabetes. (2015) 6, no. 8, 10.4239/wjd.v6.i8.1024, 1024. 26240699 PMC4515442

[5] Behboudi-Gandevani S. , Amiri M. , Bidhendi Yarandi R. , and Ramezani Tehrani F. , The Impact of Diagnostic Criteria for Gestational Diabetes on Its Prevalence: A Systematic Review and Meta-Analysis, Diabetology & Metabolic Syndrome. (2019) 11, no. 1, 10.1186/s13098-019-0406-1, 2-s2.0-85061113282. PMC6359830 30733833

[6] Al-Rifai R. H. , Abdo N. M. , Paulo M. S. , Saha S. , and Ahmed L. A. , Prevalence of Gestational Diabetes Mellitus in the Middle East and North Africa, 2000-2019: A Systematic Review, Meta-Analysis, and Meta-Regression, Frontiers in Endocrinology. (2021) 12, 10.3389/fendo.2021.668447, 668447. 34512543 PMC8427302

[7] Bashir M. M. , Ahmed L. A. , and Elbarazi I. , et al.Incidence of Gestational Diabetes Mellitus in the United Arab Emirates; Comparison of Six Diagnostic Criteria: The Mutaba’ah Study, Frontiers in Endocrinology. (2022) 13, 10.3389/fendo.2022.1069477, 1069477. 36578957 PMC9791114

[8] Vounzoulaki E. , Khunti K. , Abner S. C. , Tan B. K. , Davies M. J. , and Gillies C. L. , Progression to Type 2 Diabetes in Women With a Known History of Gestational Diabetes: Systematic Review and Meta-Analysis, British Medical Journal. (2020) 369, 10.1136/bmj.m1361, m1361. 32404325 PMC7218708

[9] Xie W. , Wang Y. , Xiao S. , Qiu L. , Yu Y. , and Zhang Z. , Association of Gestational Diabetes Mellitus With Overall and Type Specific Cardiovascular and Cerebrovascular Diseases: Systematic Review and Meta-Analysis, British Medical Journal. (2022) 378, 10.1136/bmj-2022-070244, e070244. 36130740 PMC9490552

[10] Feig D. S. , Artani A. , Asaf A. , Li P. , Booth G. L. , and Shah B. R. , Long-Term Neurobehavioral and Metabolic Outcomes in Offspring of Mothers With Diabetes During Pregnancy: A Large, Population-Based Cohort Study in Ontario, Canada, Diabetes Care. (2024) 47, no. 9, 1568–1575, 10.2337/dc24-0108. 38820461

[11] Catalano P. M. , Tyzbir E. D. , Roman N. M. , Amini S. B. , and Sims E. A. , Longitudinal Changes in Insulin Release and Insulin Resistance in Nonobese Pregnant Women, American Journal of Obstetrics and Gynecology. (1991) 165, no. 6, 1667–1672, 10.1016/0002-9378(91)90012-G, 2-s2.0-0026334046. 1750458

[12] Rieck S. and Kaestner K. H. , Expansion of β-Cell Mass in Response to Pregnancy, Trends in Endocrinology & Metabolism. (2010) 21, no. 3, 151–158, 10.1016/j.tem.2009.11.001, 2-s2.0-77949324263. 20015659 PMC3627215

[13] Buchanan T. A. and Xiang A. H. , Gestational Diabetes Mellitus, The Journal of Clinical Investigation. (2005) 115, no. 3, 485–491, 10.1172/JCI200524531, 2-s2.0-14644409707. 15765129 PMC1052018

[14] Usman T. O. , Chhetri G. , Yeh H. , and Dong H. H. , Beta-Cell Compensation and Gestational Diabetes, Journal of Biological Chemistry. (2023) 299, no. 12, 10.1016/j.jbc.2023.105405, 105405. 38229396 PMC10694657

[15] Plows J. F. , Stanley J. L. , Baker P. N. , Reynolds C. M. , and Vickers M. H. , The Pathophysiology of Gestational Diabetes Mellitus, International Journal of Molecular Sciences. (2018) 19, no. 11, 10.3390/ijms19113342, 2-s2.0-85055630359, 3342. 30373146 PMC6274679

[16] Kaufman B. A. , Li C. , and Soleimanpour S. A. , Mitochondrial Regulation of β-Cell Function: Maintaining the Momentum for Insulin Release, Molecular Aspects of Medicine. (2015) 42, 91–104, 10.1016/j.mam.2015.01.004, 2-s2.0-84939978535. 25659350 PMC4404204

[17] Georgiadou E. and Rutter G. A. , Control by Ca+^2+^ of Mitochondrial Structure and Function in Pancreatic β-Cells, Cell Calcium. (2020) 91, 10.1016/j.ceca.2020.102282, 102282. 32961506 PMC7116533

[18] Sobrevia L. , Valero P. , and Grismaldo A. , et al.Mitochondrial Dysfunction in the Fetoplacental Unit in Gestational Diabetes Mellitus, Biochimica et Biophysica Acta (BBA)-Molecular Basis of Disease. (2020) 1866, no. 12, 10.1016/j.bbadis.2020.165948, 165948. 32866635

[19] Abbade J. , Klemetti M. M. , and Farrell A. , et al.Increased Placental Mitochondrial Fusion in Gestational Diabetes Mellitus: An Adaptive Mechanism to Optimize Feto-Placental Metabolic Homeostasis?, BMJ Open Diabetes Research & Care. (2020) 8, no. 1, 10.1136/bmjdrc-2019-000923, e000923. PMC7059528 32144130

[20] Boyle K. E. , Hwang H. , and Janssen R. C. , et al.Gestational Diabetes is Characterized by Reduced Mitochondrial Protein Expression and Altered Calcium Signaling Proteins in Skeletal Muscle, PloS One. (2014) 9, no. 9, 10.1371/journal.pone.0106872, 2-s2.0-84925376523, e106872. 25216282 PMC4162568

[21] Saucedo R. , Ortega-Camarillo C. , Ferreira-Hermosillo A. , Díaz-Velázquez M. F. , Meixueiro-Calderón C. , and Valencia-Ortega J. , Role of Oxidative Stress and Inflammation in Gestational Diabetes Mellitus, Antioxidants. (2023) 12, no. 10, 10.3390/antiox12101812, 1812. 37891891 PMC10604289

[22] Lin X.-J. , Xu X.-X. , and Zhang H.-X. , et al.Placental mtDNA Copy Number and Methylation in Association With Macrosomia in Healthy Pregnancy, Placenta. (2022) 118, 1–9, 10.1016/j.placenta.2021.12.021. 34972066

[23] Pickles S. , Vigié P. , and Youle R. J. , Mitophagy and Quality Control Mechanisms in Mitochondrial Maintenance, Current Biology. (2018) 28, no. 4, R170–R85, 10.1016/j.cub.2018.01.004, 2-s2.0-85042076557. 29462587 PMC7255410

[24] Marinković M. and Novak I. , A Brief Overview of BNIP3L/NIX Receptor-Mediated Mitophagy, FEBS Open Bio. (2021) 11, no. 12, 3230–3236, 10.1002/2211-5463.13307. PMC8634856 34597467

[25] Yun J. , Puri R. , and Yang H. , et al.MUL1 Acts in Parallel to the PINK1/Parkin Pathway in Regulating Mitofusin and Compensates for Loss of PINK1/Parkin, Elife. (2014) 3, 10.7554/eLife.01958, e01958. 24898855 PMC4044952

[26] Wu H. , Wang Y. , and Li W. , et al.Deficiency of Mitophagy Receptor FUNDC1 Impairs Mitochondrial Quality and Aggravates Dietary-Induced Obesity and Metabolic Syndrome, Autophagy. (2019) 15, no. 11, 1882–1898, 10.1080/15548627.2019.1596482, 2-s2.0-85063957702. 30898010 PMC6844496

[27] Jones H. N. , Woollett L. A. , Barbour N. , Prasad P. D. , Powell T. L. , and Jansson T. , High-Fat Diet before and During Pregnancy Causes Marked up-Regulation of Placental Nutrient Transport and Fetal Overgrowth in C57/BL6 Mice, The FASEB Journal. (2009) 23, no. 1, 271–278, 10.1096/fj.08-116889, 2-s2.0-58249102098. 18827021 PMC2626621

[28] Abu Samra N. , Jelinek H. F. , and Alsafar H. , et al.Genomics and Epigenomics of Gestational Diabetes Mellitus: Understanding the Molecular Pathways of the Disease Pathogenesis, International Journal of Molecular Sciences. (2022) 23, no. 7, 10.3390/ijms23073514, 3514. 35408874 PMC8998752

[29] Huang T.-T. , Sun W.-J. , Liu H.-Y. , Ma H.-L. , and Cui B.-X. , p66Shc-Mediated Oxidative Stress is Involved in Gestational Diabetes Mellitus, World Journal of Diabetes. (2021) 12, no. 11, 1894–1907, 10.4239/wjd.v12.i11.1894. 34888014 PMC8613666

[30] Ji L. , Zhang X. , and Chen Z. , et al.High Glucose-Induced p66Shc Mitochondrial Translocation Regulates Autophagy Initiation and Autophagosome Formation in Syncytiotrophoblast and Extravillous Trophoblast, Cell Communication and Signaling. (2024) 22, no. 1, 10.1186/s12964-024-01621-x. PMC11031965 38643181

[31] Al Bekai E. , Beaini C. E. , and Kalout K. , et al.The Hidden Impact of Gestational Diabetes: Unveiling Offspring Complications and Long-Term Effects, Life. (2025) 15, no. 3, 10.3390/life15030440. PMC11944258 40141785

[32] Hjort L. , Novakovic B. , and Grunnet L. G. , et al.Diabetes in Pregnancy and Epigenetic Mechanisms—How the First 9 Months From Conception Might Affect the Child’s Epigenome and Later Risk of Disease, The Lancet Diabetes & Endocrinology. (2019) 7, no. 10, 796–806, 10.1016/S2213-8587(19)30078-6, 2-s2.0-85072195647. 31128973

[33] Słupecka-Ziemilska M. , Wychowański P. , and Puzianowska-Kuznicka M. , Gestational Diabetes Mellitus Affects Offspring’s Epigenome. Is There a Way to Reduce the Negative Consequences?, Nutrients. (2020) 12, no. 9, 10.3390/nu12092792, 2792. 32933073 PMC7551316

[34] Wang N. , Li S. , and Yang L. , DNA Methylation Patterns and Predictive Models for Metabolic Disease Risk in Offspring of Gestational Diabetes Mellitus, Diabetology & Metabolic Syndrome. (2025) 17, no. 1, 10.1186/s13098-025-01707-7. PMC12046688 40312441

[35] He F. , Huang Y. , and Song Z. , et al.Mitophagy-Mediated Adipose Inflammation Contributes to Type 2 Diabetes With Hepatic Insulin Resistance, Journal of Experimental Medicine. (2020) 218, no. 3, 10.1084/jem.20201416, e20201416. PMC7927432 33315085

[36] de Maranon A. M. , Díaz-Pozo P. , and Canet F. , et al.Metformin Modulates Mitochondrial Function and Mitophagy in Peripheral Blood Mononuclear Cells From Type 2 Diabetic Patients, Redox Biology. (2022) 53, 10.1016/j.redox.2022.102342, 102342. 35605453 PMC9124713

[37] Wang T. , Wang X. , and Fu T. , et al.Roles of Mitochondrial Dynamics and Mitophagy in Diabetic Myocardial Microvascular Injury, Cell Stress and Chaperones. (2023) 28, no. 6, 675–688, 10.1007/s12192-023-01384-3. 37755621 PMC10746668

[38] Hasson S. A. , Kane L. A. , and Yamano K. , et al.High-Content Genome-Wide RNAi Screens Identify Regulators of Parkin Upstream of Mitophagy, Nature. (2013) 504, no. 7479, 291–295, 10.1038/nature12748, 2-s2.0-84890429468. 24270810 PMC5841086

[39] Pickrell A. M. and Youle R. J. , The Roles of PINK1, Parkin, and Mitochondrial Fidelity in Parkinson’s Disease, Neuron. (2015) 85, no. 2, 257–273, 10.1016/j.neuron.2014.12.007, 2-s2.0-84921369563. 25611507 PMC4764997

[40] Jiang D. , Cui H. , and Xie N. , et al.ATF4 Mediates Mitochondrial Unfolded Protein Response in Alveolar Epithelial Cells, American Journal of Respiratory Cell and Molecular Biology. (2020) 63, no. 4, 478–489, 10.1165/rcmb.2020-0107OC. 32551949 PMC7528926

[41] Quirós P. M. , Prado M. A. , and Zamboni N. , et al.Multi-Omics Analysis Identifies ATF4 as a Key Regulator of the Mitochondrial Stress Response in Mammals, Journal of Cell Biology. (2017) 216, no. 7, 2027–2045, 10.1083/jcb.201702058, 2-s2.0-85021857064. 28566324 PMC5496626

[42] Aghaeepour N. , Ganio E. A. , and McIlwain D. , et al.An Immune Clock of Human Pregnancy, Science Immunology. (2017) 2, no. 15, 10.1126/sciimmunol.aan2946, 2-s2.0-85042607653, eaan2946. 28864494 PMC5701281

[43] Tang Z. , Wang S. , and Li X. , et al.Longitudinal Integrative Cell-Free DNA Analysis in Gestational Diabetes Mellitus, Cell Reports Medicine. (2024) 5, no. 8, 10.1016/j.xcrm.2024.101660, 101660. 39059385 PMC11384941

[44] Lewis K. A. , Chang L. , and Cheung J. , et al.Systematic Review of Transcriptome and microRNAome Associations With Gestational Diabetes Mellitus, Frontiers in Endocrinology. (2022) 13, 10.3389/fendo.2022.971354, 971354. 36704034 PMC9871895

[45] Cheng Z. , FoxO Transcription Factors in Mitochondrial Homeostasis, Biochemical Journal. (2022) 479, no. 4, 525–536, 10.1042/BCJ20210777. 35195252 PMC8883485

[46] Mammucari C. , Milan G. , and Romanello V. , et al.FoxO3 Controls Autophagy in Skeletal Muscle in Vivo, Cell Metabolism. (2007) 6, no. 6, 458–471, 10.1016/j.cmet.2007.11.001, 2-s2.0-36448940798. 18054315

[47] Dodson M. , Shakya A. , Anandhan A. , Chen J. , Garcia J. G. N. , and Zhang D. D. , NRF2 and Diabetes: The Good, the Bad, and the Complex, Diabetes. (2022) 71, no. 12, 2463–2476, 10.2337/db22-0623. 36409792 PMC9750950

[48] Luchkova A. , Mata A. , and Cadenas S. , Nrf2 as a Regulator of Energy Metabolism and Mitochondrial Function, FEBS Letters. (2024) 598, no. 17, 2092–2105, 10.1002/1873-3468.14993. 39118293

[49] Lu H. , Wang X. , and Li M. , et al.Mitochondrial Unfolded Protein Response and Integrated Stress Response as Promising Therapeutic Targets for Mitochondrial Diseases, Cells. (2022) 12, no. 1, 10.3390/cells12010020. PMC9818186 36611815

[50] Hebert J. F. and Myatt L. , Placental Mitochondrial Dysfunction With Metabolic Diseases: Therapeutic Approaches, Biochimica et Biophysica Acta (BBA)-Molecular Basis of Disease. (2021) 1867, no. 1, 10.1016/j.bbadis.2020.165967, 165967. 32920120 PMC8043619

[51] Yan Y.-S. , Feng C. , and Yu D.-Q. , et al.Long-Term Outcomes and Potential Mechanisms of Offspring Exposed to Intrauterine Hyperglycemia, Frontiers in Nutrition. (2023) 10, 10.3389/fnut.2023.1067282, 1067282. 37255932 PMC10226394

[52] Castillo-Castrejon M. and Powell T. L. , Placental Nutrient Transport in Gestational Diabetic Pregnancies, Frontiers in Endocrinology. (2017) 8, 10.3389/fendo.2017.00306, 2-s2.0-85034054091. PMC5682011 29163373

[53] Chen Y. , Liu X. , and Liu Y. , et al.Mitochondrial Quality Control in Diabetes Mellitus and Complications: Molecular Mechanisms and Therapeutic Strategies, Cell Death & Disease. (2025) 16, no. 1, 10.1038/s41419-025-07936-y. PMC12391366 40866350

[54] Ni D. and Nanan R. , Elucidating the Maternal and Fetal Metabolic and Immune Landscapes of Gestational Diabetes Mellitus With a Pan-Organ Transcriptomic Atlas, Genes & Diseases. (2025) 13, no. 1, 10.1016/j.gendis.2025.101551, 101551. 41049131 PMC12495274

[55] Barrozo E. R. , Racusin D. A. , and Jochum M. D. , et al.Discrete Placental Gene Expression Signatures Accompany Diabetic Disease Classifications During Pregnancy, American Journal of Obstetrics and Gynecology. (2025) 232, no. 3, 326.e1–326.e15, 10.1016/j.ajog.2024.05.014. 38763341

