# Supplementary material for: Disruption of Mitophagy‐Related Gene Expression in Gestational Diabetes Mellitus: A Transcriptomic and Machine Learning Approach
Source: J Diabetes Res. 2026 Mar 24;2026:7913374. doi: 10.1155/jdr/7913374 (PMC13140379; doi:10.1155/jdr/7913374)
Supplement: Supplementary file 1 — Supporting Information A Word document containing these supporting items is provided alongside the manuscript. The supporting information are described below in brief: (I) Supplementary Methods: The Supplementary Methods outline the advanced analyses conducted to support the primary results. We begin by validating trimester‐specific classifiers through repeated, stratified subsampling (100 iterations) to evaluate sampling stability beyond cross‐validation, reporting mean and SD values for accuracy, recall, and class‐probability margins. Next, we conduct unsupervised coexpression analysis of mitophagy genes (using Ward.D2/Euclidean clustering) and visualize the results with heat maps to identify trimester‐ and condition‐specific patterns. Temporal gene dynamics are modeled using generalized additive models (GAMs) across gestational age to capture nonlinear expression trends (e.g., PINK1, ATF4, and TOMM7). Upstream regulatory mechanisms are investigated through iRegulon motif enrichment, which predicts transcription factors targeting downregulated mitophagy genes, with TF→target networks visualized for clarity. Crosstalk analyses (using crosstalkR and KEGG graphs) examine intersections between mitophagy pathways and insulin/metabolic‐stress signaling. The mitochondrial localization of key genes is verified using the Human Protein Atlas. Lastly, batch effects are assessed (via SVA and PCA), confirming no significant batch‐related variance prior to downstream analyses. (II) Supporting Figures: Figure S1. A KEGG schematic of the mitophagy pathway is used as a reference framework. Figure S2. A heat map showing the expression levels of mitophagy‐related genes in placental samples (GDM versus control), pooled from GSE203346 and GSE154414 (n = 92). Highlighted are key downregulated genes (PINK1, TOMM7, ATF4, and MUL1). Figure S3. A GO enrichment analysis of MUL1 interactors using ClueGO: bar charts (biological processes and molecular functions) and pie charts (category distr [file JDR-2026-7913374-s001.docx]

Supplementary Material

Disruption of Mitophagy-Related Gene Expression in Gestational Diabetes Mellitus: A Transcriptomic and Machine Learning Approach

Souhaib Bouati et al.

1. **Supplementary Methods:**

**1. Classifier validation and sampling stability:**

To assess the performance of the multiclassPairs classifier beyond cross-validation, random stratified subsampling was conducted across trimesters for both control and GDM samples in GSE154377. Sampling was repeated 100 times, with performance metrics recorded for each iteration. Consistency across runs was evaluated through the calculation of average values and standard deviations for accuracy, recall, and class probability margins.

**2. Gene co-expression clustering:**

Unsupervised hierarchical clustering of mitophagy genes was performed using the Ward.D2 method with Euclidean distance on normalized expression counts (TPM values). Clustering patterns were visualized through heatmaps generated with the ComplexHeatmap package, illustrating trimester-specific trends for both GDM and control groups.

**3. Temporal modeling of gene dynamics:**

We employed generalized additive models (GAMs), implemented using the mgcv package in R, to model the temporal dynamics of key mitophagy genes, including PINK1, ATF4, and TOMM7. Gene expressions were analyzed as a function of gestational age (in weeks) through penalized regression splines, enabling the identification of nonlinear trends in samples from gestational diabetes mellitus (GDM) versus control groups.

**4. Transcription factor binding prediction:**

We utilized the iRegulon plugin in Cytoscape to investigate upstream regulatory mechanisms of dysregulated MRGs by identifying transcription factors targeting downregulated MRGs. Transcription factor motif enrichment was assessed using NES scores, and network plots were generated to illustrate TF-gene interactions.

**5. Pathway crosstalk analysis:**

The crosstalkR tool was employed to identify shared and distinct signaling crosstalk between mitophagy-related pathways and insulin signaling/metabolic stress pathways. Additionally, KEGG-based graphs were generated to visually represent interactions among differentially expressed pathways.

**6. Mitochondrial localization confirmation:**

We validated subcellular localization by cross-referencing the Human Protein Atlas (HPA) database for the downregulated MRGs. Genes with experimentally verified mitochondrial localization, such as PINK1, MUL1, and TOMM7, were annotated in Supplementary Table S1.

**7. Technical batch correction:**

GSE154377 employed Surrogate Variable Analysis (SVA) to evaluate latent batch effects, considering its longitudinal design. Principal Component Analysis (PCA) revealed no substantial batch-related variance, and thus, no corrections were implemented prior to downstream analyses.

1. **Supplementary Figures:**

**
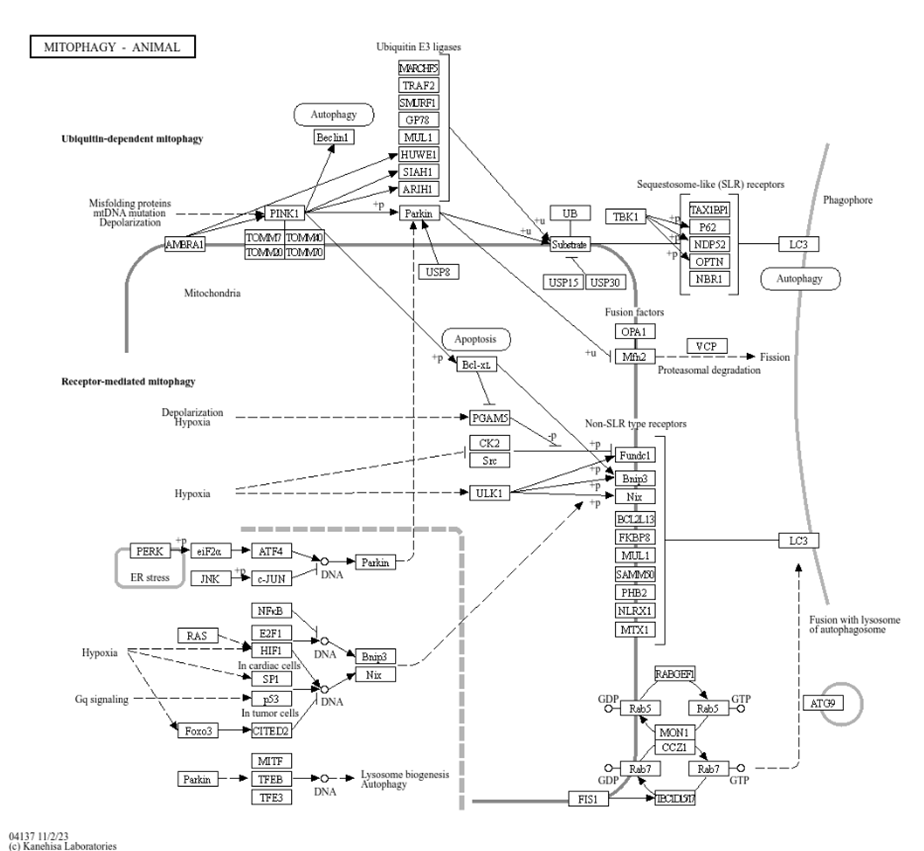
**

**Supplementary Figure 1. Mitophagy pathway as depicted in the KEGG database.**


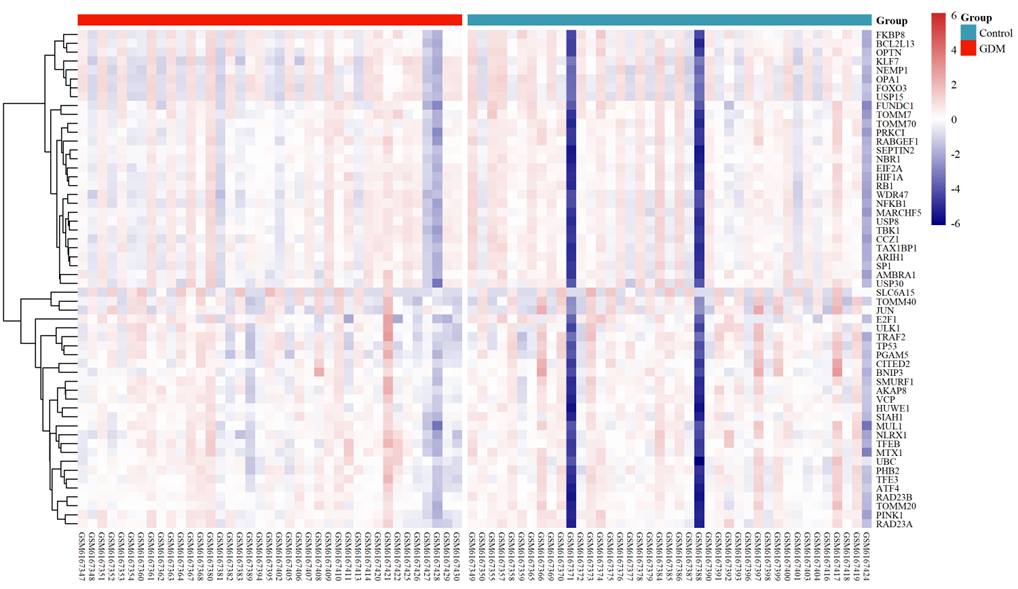


**Supplementary Figure S2. *The expression of mitophagy-related genes in placental samples from GDM patients and controls*.** Heatmap of mitophagy-related genes in placentas from GDM and control pregnancy. Values are variance-stabilized and row-scaled (Z-scores). Warmer colors indicate higher expression; cooler colors indicate lower expression. The columns are samples (grouped), and the rows are genes; both were hierarchically clustered (Euclidean distance, complete linkage). GSE203346 and GSE154414 are combined (pooled n = 92); key downregulated genes (*PINK1*, *TOMM7*, *ATF4*, *MUL1*) are highlighted.


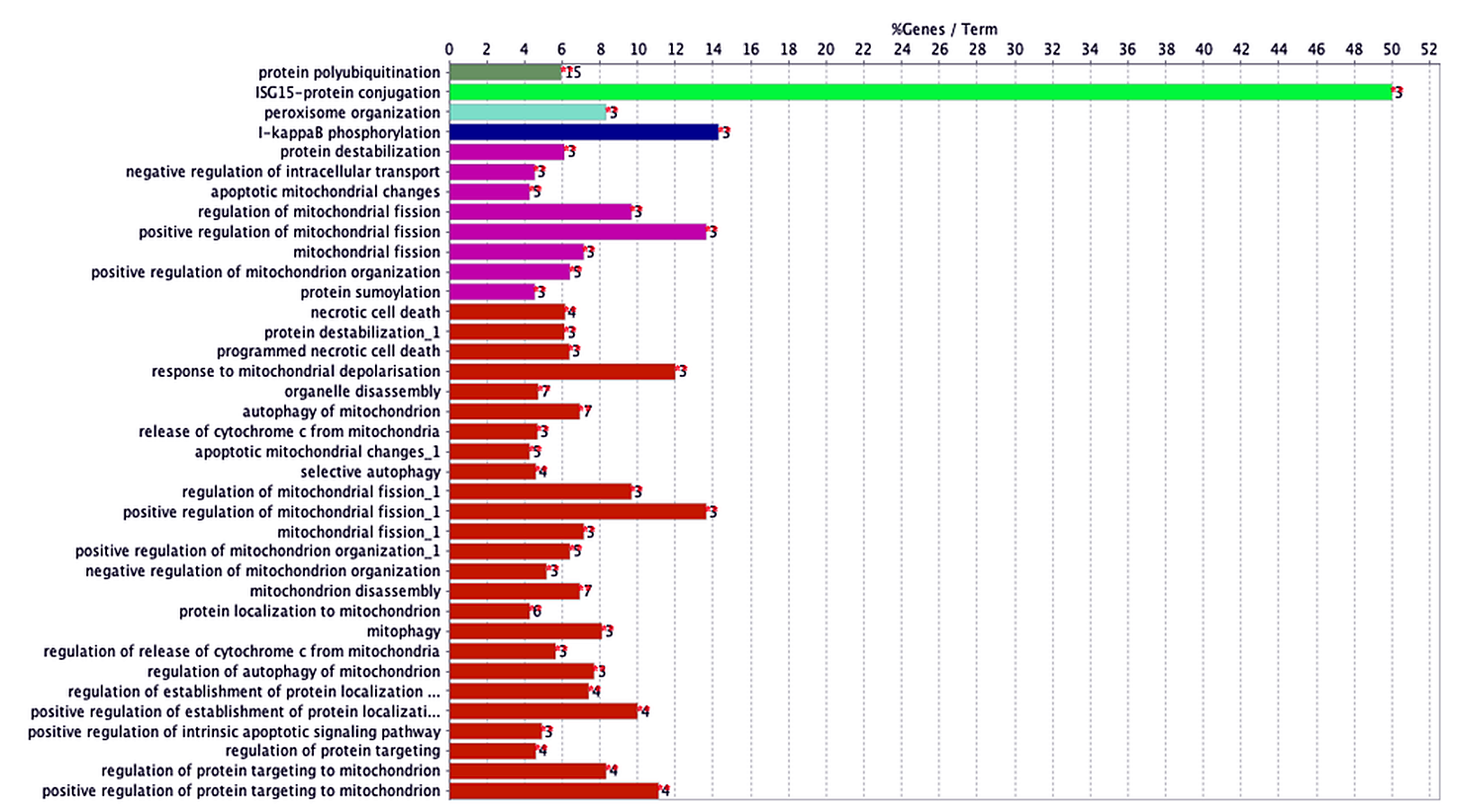


**(A)**


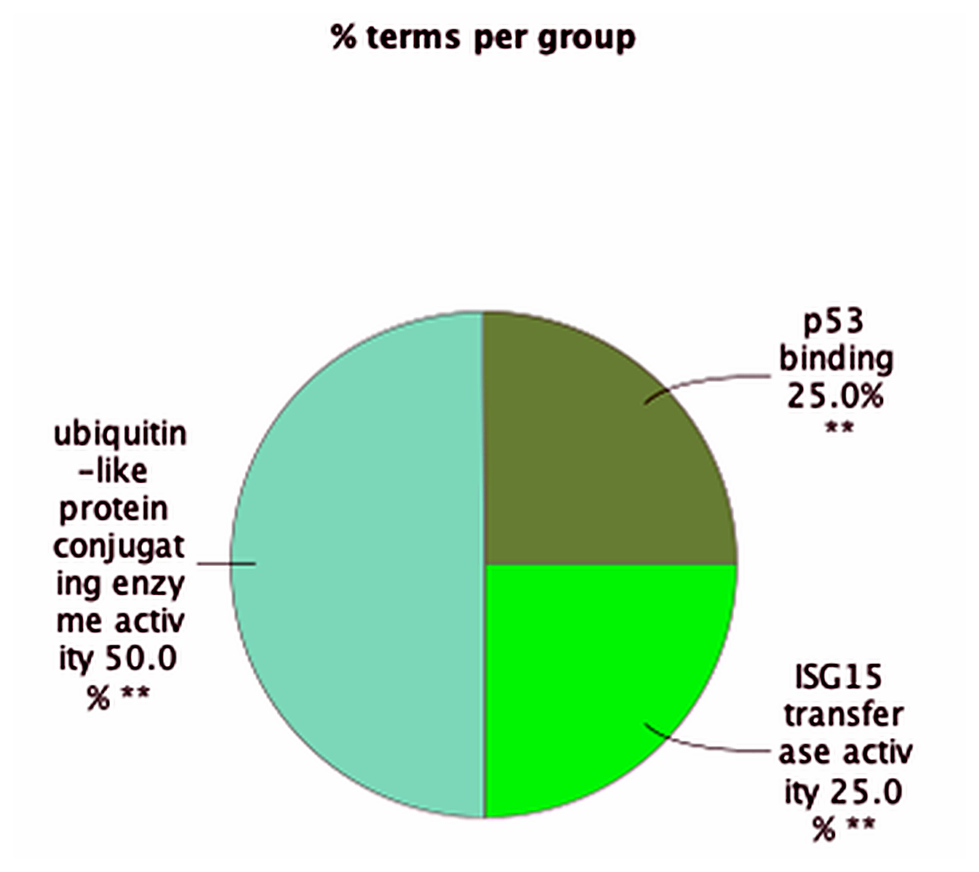


**(B)**


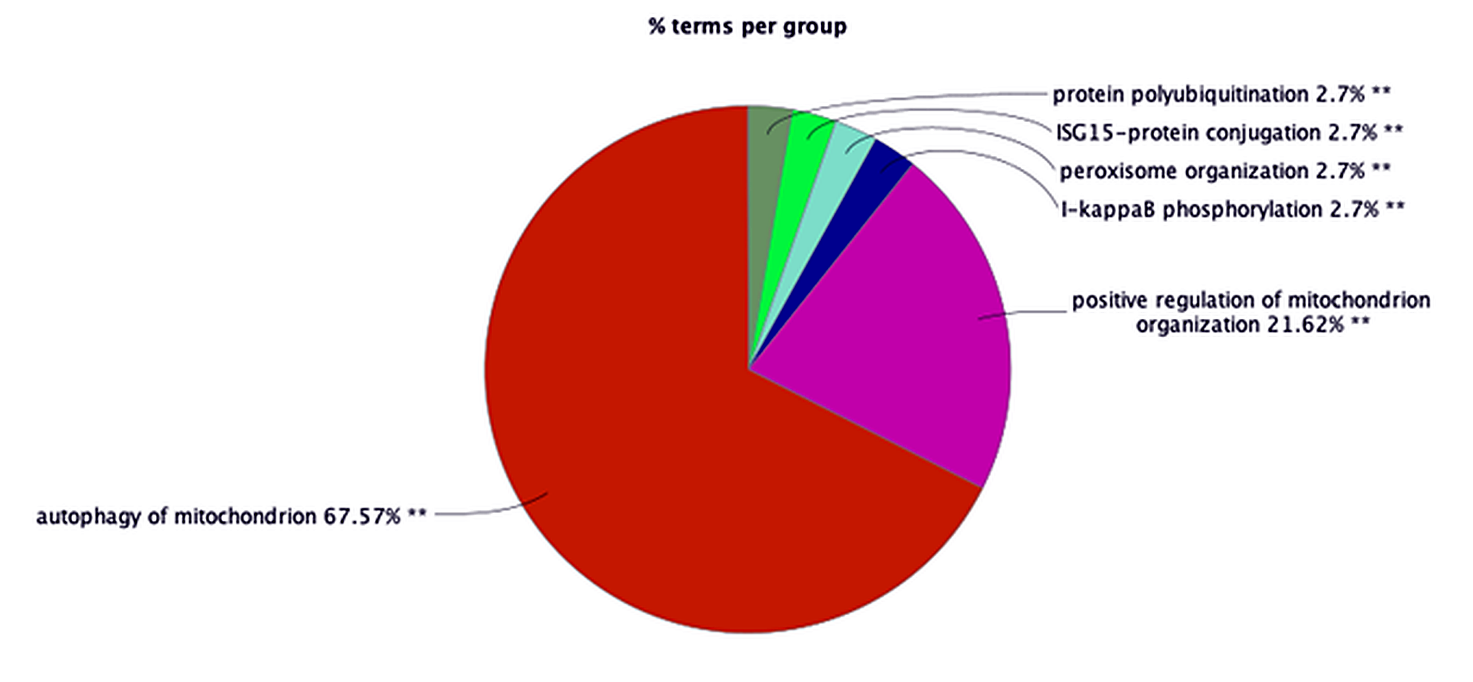


**(C)**

**Supplementary Figure S3. *GO enrichment analysis of MUL1 interactors***. The selection of MUL1 for this detailed enrichment analysis was based on the similar functional interactions displayed by all genes. ClueGO plugin was used to obtain enrichment pathways (P < 0.05). Enrichment based on biological processes and molecular functions (MF) can be seen in bar charts (A) and pie charts (B) and (C). The major molecular functions identified include p53 binding, ISG15 transferase activity, ubiquitin-like protein conjugating enzyme activity, and ubiquitin conjugating enzyme activity, suggesting roles in protein modification processes such as ubiquitination, ISGylation, and protein stability regulation. We found no significantly enriched cellular component (CC) terms (P < 0.05). The selection of MUL1 for this detailed enrichment analysis was based on the similar functional interactions displayed by all genes. ClueGO plugin was used to obtain enrichment pathways (P < 0.05).


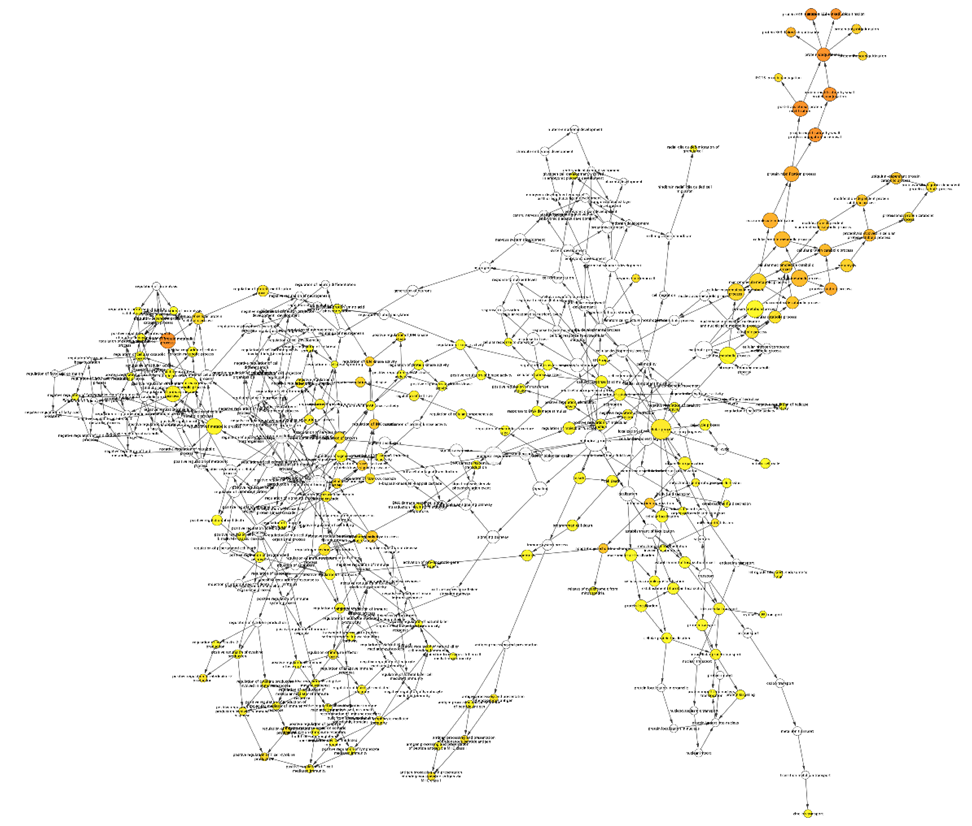


**Supplementary Figure S4.** Analyses of functional enrichment for the overarching protein–protein interaction (PPI) network that integrates target proteins with their interacting partners. Gene Ontology (GO) terms and pathways significantly enriched in this network are listed in the figures below.


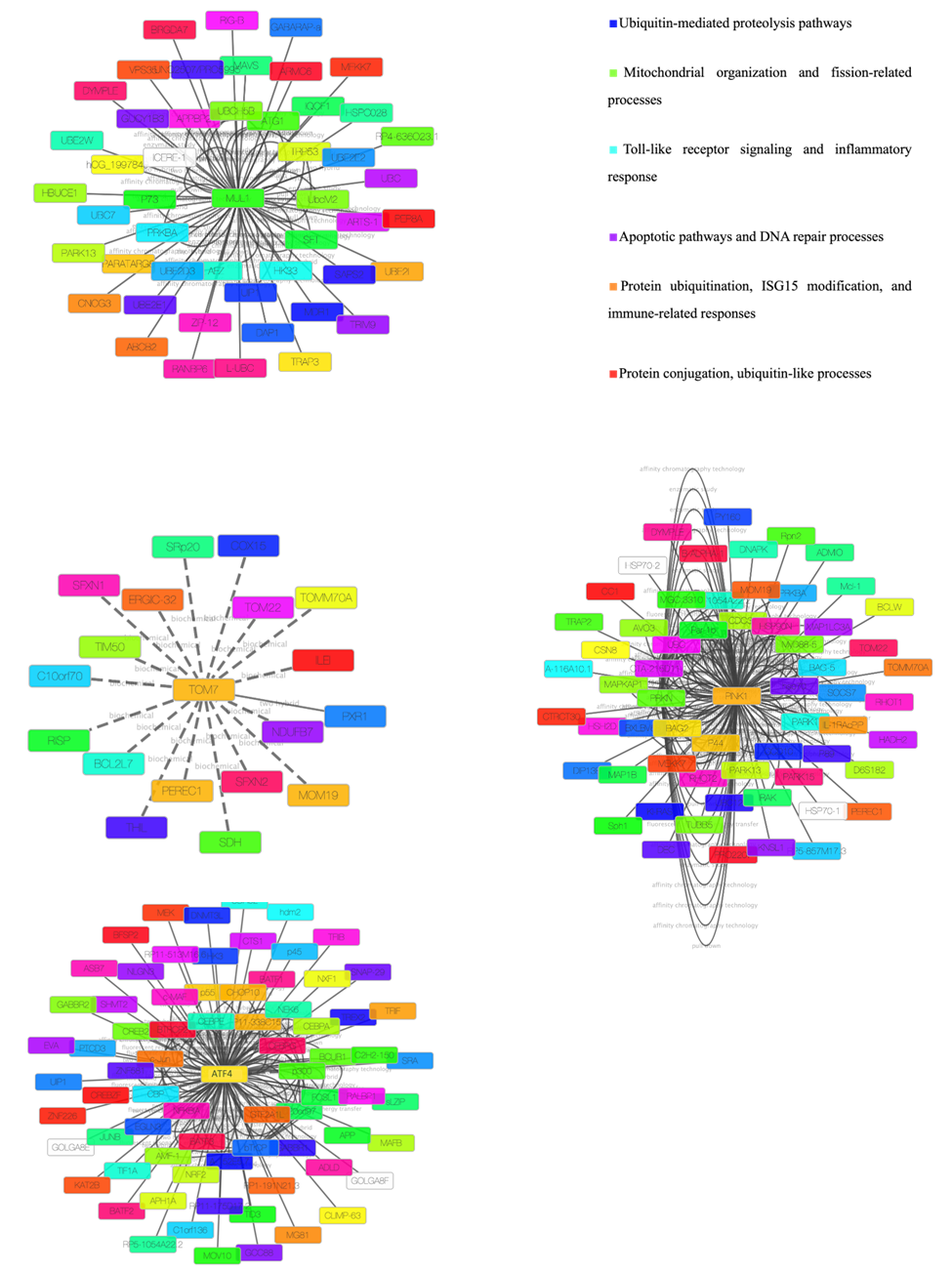


**(D)**

**(C)**

**(B)**

**(A)**

**Supplementary Figure S5. *A PPI network illustrates the interaction partners of (A) MUL1, (B) TOM7, (C) PINK1, and (D) ATF4***. Networks were generated using STRING. Colors on nodes correspond to Gene Ontology (GO) functional categories, highlighting different biological roles.


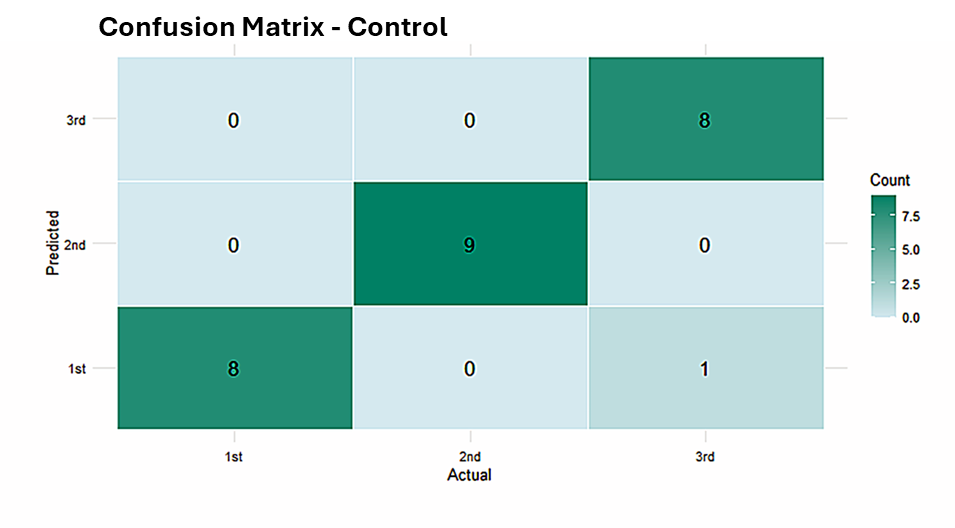


**(A)**

**(B)**


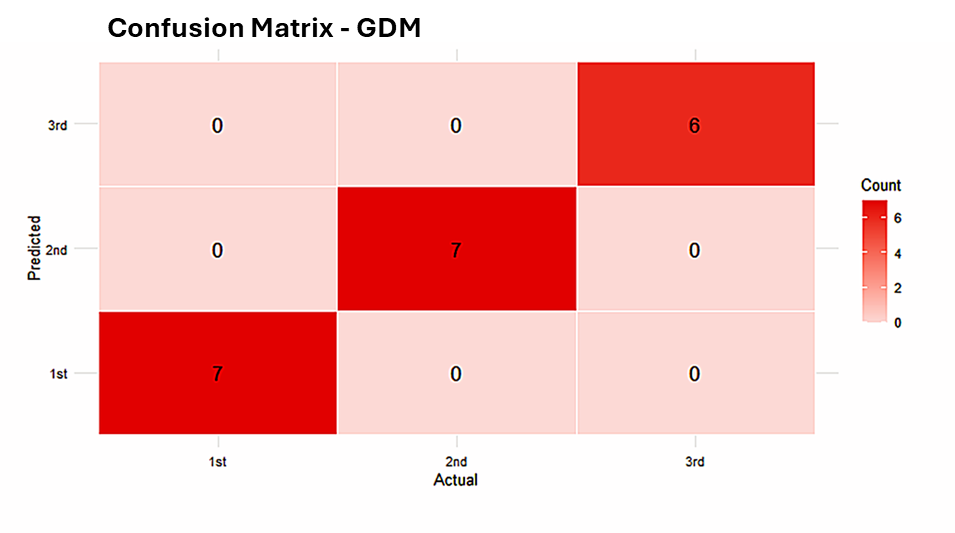


**Supplementary Figure S6. *A trimester-specific classifier for control and GDM samples was derived using the multiclassPairs R package with the GSE154377 dataset***. We subset RNA-seq data from control and gestational diabetes mellitus (GDM) placental samples by trimester (1st, 2nd, and 3rd), and then the data was variance-stabilized transformed (VST). A trimester-specific classifier was trained for the control group (A) and for the GDM group (B) using mitochondrial genes present in both datasets. The one-vs-rest filter (100 top features, UpDown = TRUE) was used to select features, and the train_one_vs_rest_TSP function was used to train and evaluate models.

1. **Supplementary Tables:**

Supplementary Table 1: Genes associated with mitophagy included in the current study and their differential expression results

| **ID (Entrez**) | **Gene Symbol** | **logFC** | **logCPM** | **P-Value** | **P-value (FDR)** |
| --- | --- | --- | --- | --- | --- |
| 79594 | *MUL1* | -0.955 | 10.719 | 4.82E-06 | 2.08E-04 |
| 65018 | *PINK1* | -0.472 | 13.446 | 6.94E-05 | 2.01E-04 |
| 54543 | *TOMM7* | -0.496 | 13.182 | 6.75E-04 | 1.30E-02 |
| 468 | *ATF4* | -0.659 | 14.690 | 1.06E-03 | 1.53E-02 |
| 139341 | *FUNDC1* | -0.381 | 10.445 | 2.23E-03 | 2.59E-02 |
| 3091 | *HIF1A* | 0.386 | 14.209 | 1.10E-02 | 9.54E-02 |
| 1869 | *E2F1* | -0.446 | 9.369 | 1.15E-02 | 9.54E-02 |
| 51024 | *FIS1* | -0.451 | 14.635 | 1.40E-02 | 9.73E-02 |
| 79671 | *NLRX1* | 0.319 | 9.976 | 1.68E-02 | 9.73E-02 |
| 27342 | *RABGEF1* | 0.277 | 12.559 | 1.68E-02 | 9.73E-02 |
| 5925 | *RB1* | 0.302 | 13.643 | 2.80E-02 | 1.48E-01 |
| 25820 | *ARIH1* | 0.430 | 14.269 | 3.92E-02 | 1.71E-01 |
| 9101 | *USP8* | 0.322 | 13.730 | 3.99E-02 | 1.71E-01 |
| 664 | *BNIP3* | 0.500 | 11.979 | 4.14E-02 | 1.71E-01 |
| 22911 | *WDR47* | 0.265 | 11.617 | 4.15E-02 | 1.71E-01 |
| 7316 | *UBC* | 0.456 | 17.018 | 5.21E-02 | 1.81E-01 |
| 4735 | *SEPTIN2* | 0.265 | 14.912 | 5.31E-02 | 1.81E-01 |
| 4790 | *NFKB1* | 0.324 | 13.198 | 7.09E-02 | 2.29E-01 |
| 6667 | *SP1* | 0.335 | 14.735 | 8.46E-02 | 2.58E-01 |
| 51622 | *CCZ1* | 0.202 | 12.378 | 9.89E-02 | 2.87E-01 |
| 7186 | *TRAF2* | -0.354 | 10.285 | 1.55E-01 | 3.19E-01 |
| 9868 | *TOMM70* | 0.152 | 12.753 | 1.22E-01 | 3.12E-01 |
| 10075 | *HUWE1* | 0.266 | 15.329 | 1.40E-01 | 3.52E-01 |
| 5584 | *PRKCI* | 0.126 | 11.550 | 1.49E-01 | 3.61E-01 |
| 5887 | *RAD23B* | -0.162 | 14.535 | 1.61E-01 | 3.65E-01 |
| 8609 | *KLF7* | 0.467 | 13.816 | 1.63E-01 | 3.65E-01 |
| 84749 | *USP30* | 0.163 | 10.178 | 1.86E-01 | 4.00E-01 |
| 5886 | *RAD23A* | -0.161 | 12.949 | 2.48E-01 | 5.14E-01 |
| 4077 | *NBR1* | 0.139 | 14.840 | 2.63E-01 | 5.18E-01 |
| 55117 | *SLC6A15* | -0.563 | 8.073 | 2.69E-01 | 5.18E-01 |
| 8408 | *ULK1* | 0.307 | 13.376 | 2.77E-01 | 5.18E-01 |
| 11331 | *PHB2* | -0.182 | 12.701 | 3.24E-01 | 5.87E-01 |
| 6477 | *SIAH1* | -0.130 | 13.303 | 3.51E-01 | 6.13E-01 |
| 55626 | *AMBRA1* | 0.102 | 12.459 | 3.59E-01 | 6.13E-01 |
| 9958 | *USP15* | 0.345 | 16.695 | 3.95E-01 | 6.28E-01 |
| 7415 | *VCP* | -0.140 | 14.121 | 3.96E-01 | 6.28E-01 |
| 83939 | *EIF2A* | 0.086 | 13.126 | 4.02E-01 | 6.28E-01 |
| 23770 | *FKBP8* | -0.238 | 16.888 | 4.11E-01 | 6.28E-01 |
| 9804 | *TOMM20* | -0.097 | 13.618 | 4.34E-01 | 6.30E-01 |
| 10370 | *CITED2* | 0.195 | 13.324 | 4.34E-01 | 6.30E-01 |
| 7942 | *TFEB* | 0.134 | 11.145 | 4.46E-01 | 6.32E-01 |
| 192111 | *PGAM5* | -0.136 | 10.591 | 5.02E-01 | 6.93E-01 |
| 10133 | *OPTN* | -0.122 | 14.387 | 5.55E-01 | 7.48E-01 |
| 10452 | *TOMM40* | -0.143 | 11.025 | 6.23E-01 | 8.21E-01 |
| 2309 | *FOXO3* | 0.178 | 17.136 | 6.78E-01 | 8.71E-01 |
| 3725 | *JUN* | 0.182 | 12.961 | 6.91E-01 | 8.71E-01 |
| 6714 | *c-Src* | 0.114 | 11.014 | 7.12E-01 | 8.79E-01 |
| 57154 | *SMURF1* | -0.046 | 12.687 | 7.75E-01 | 9.10E-01 |
| 7157 | *TP53* | -0.050 | 11.581 | 7.76E-01 | 9.10E-01 |
| 54708 | *MARCHF5* | 0.075 | 11.902 | 7.86E-01 | 9.10E-01 |
| 8887 | *TAX1BP1* | 0.052 | 15.204 | 8.01E-01 | 9.10E-01 |
| 4580 | *MTX1* | 0.044 | 11.505 | 8.27E-01 | 9.16E-01 |
| 7030 | *TFE3* | -0.016 | 12.844 | 8.37E-01 | 9.16E-01 |
| 23786 | *BCL2L13* | 0.014 | 13.434 | 8.85E-01 | 9.32E-01 |
| 4976 | *OPA1* | 0.035 | 15.911 | 8.96E-01 | 9.32E-01 |
| 10270 | *AKAP8* | -0.001 | 12.558 | 9.00E-01 | 9.32E-01 |
| 23306 | *NEMP1* | -0.001 | 12.161 | 9.37E-01 | 9.53E-01 |
| 29110 | *TBK1* | 0.026 | 12.835 | 9.93E-01 | 9.93E-01 |

Abbreviations: ID: Entrez IDs; LogFC: Log fold change, logCPM: Log counts per million; FDR: False discovery ration.

Supplementary Table S2: The bioinformatics and analysis tools used in this study

| **Tool/Package** | **Purpose** | **Source/Website** |
| --- | --- | --- |
| GEO Database | Dataset retrieval | <https://www.ncbi.nlm.nih.gov/geo/> |
| edgeR | Differential gene expression analysis (normalization, filtering, dispersion estimation, DEG detection) | Bioconductor |
| pheatmap | Heatmap generation (mitophagy gene expression visualization) | R package |
| ggplot2 | Visualization (Volcano, Dot, Bar plots, PCA plots) | R package |
| Cytoscape | PPI network visualization | <https://cytoscape.org/> |
| ClueGO | GO and pathway enrichment analysis in Cytoscape | Cytoscape plugin |
| BioGRID | Protein–protein interaction data source | <https://thebiogrid.org/> |
| biomaRt | Gene ID conversion (Ensembl to Entrez) | Bioconductor |
| org.Hs.eg.db | Gene ID annotation and mapping (Entrez ID retrieval) | Bioconductor |
| data.table | Data loading, sample renaming, dataset merging, preprocessing | R package |
| AnnotationDbi | Gene ID mapping support | Bioconductor |

Supplementary Table S3: Full description of the Key mitophagy-related genes

| **Official full name** | **Official symbol** | **Gene ID** | **Location** | **Function** |
| --- | --- | --- | --- | --- |
| Mitochondrial E3 ubiquitin protein ligase 1 | **MUL1** | 79594 | 1p36.12 | Encodes an E3 ubiquitin ligase involved in mitochondrial dynamics. It promotes mitophagy, regulates mitochondrial fission, and can act as a pro-apoptotic factor under cellular stress conditions. Important for mitochondrial quality control and apoptosis. |
| PTEN induced kinase 1 | **PINK1** | 65018 | 1p36.12 | Encodes a mitochondrial serine/threonine-protein kinase that protects cells from stress-induced mitochondrial dysfunction. PINK1 is crucial for initiating mitophagy by recruiting Parkin to damaged mitochondria, playing a key role in mitochondrial quality control. |
| Translocase of outer mitochondrial membrane 7 | **TOMM7** | 54543 | 7q22.1 | Encodes a component of the translocase of the outer mitochondrial membrane (TOM) complex. TOMM7 is involved in the import of proteins into mitochondria and has a regulatory role in mitochondrial membrane potential maintenance and mitophagy activation. |
| Activating transcription factor 4 | **ATF4** | 468 | 22q13.1 | Encodes a transcription factor activated by various cellular stresses, particularly endoplasmic reticulum (ER) stress. ATF4 regulates genes involved in amino acid metabolism, oxidative stress response, apoptosis, and has emerging roles in β-cell survival and diabetes pathophysiology. |

Supplementary Table S4. Performance of trimester classifiers on GSE154377 (Control vs GDM).

|  |  | **Macro** | | | **Weighted** | | |
| --- | --- | --- | --- | --- | --- | --- | --- |
| Cohort | Accuracy | Precision (macro) | Recall (macro) | F1 (macro) | Precision (weighted) | Recall (weighted) | F1 (weighted) |
| Control | 0.962 | 0.963 | 0.963 | 0.961 | 0.966 | 0.962 | 0.962 |
| GDM | 1.000 | 1.000 | 1.000 | 1.000 | 1.000 | 1.000 | 1.000 |

This table summarizes the performance of the classifier on GSE154377. The three-class trimester model performed well with controls (accuracy 0.962; macro precision/recall/F1 0.963/0.963/0.961; weighted precision/recall/F1 0.966/0.962/0.962). Under both macro and weighted averaging, accuracy, precision, recall, and F1 for the GDM cohort were 1.000, indicating perfect agreement between predictions and annotated trimester labels.
